# Investigating Neotenic and Metamorphic Axolotl Brain Complexity: A Stereological and Immunohistochemical Perspective

**DOI:** 10.1002/cne.70031

**Published:** 2025-03-20

**Authors:** Arife Ahsen Kaplan, Gürkan Öztürk, Sadık Bay, İlknur Keskin

**Affiliations:** ^1^ Department of Histology and Embryology The School of Medicine, Istanbul Medipol University Istanbul Turkey; ^2^ Regenerative and Restorative Medicine Research Center (REMER), Institute for Health Sciences and Technologies (SABITA) İstanbul Medipol University Istanbul Turkey; ^3^ Department of Physiology, School of Medicine Bolu Abant İzzet Baysal Üniversitesi Bolu Turkey

**Keywords:** axolotl, ependymoglia, metamorphosis, RRID:AGSC_100A, stereology, telencephalon

## Abstract

The ability of certain tetrapods, such as amphibians, to regenerate complex structures, such as organs or limbs, is well‐established, though this capacity varies significantly across species, with humans exhibiting limited regenerative potential. Ependymoglia cells in the ventricular region of the brain are known to exhibit proliferative properties during homeostasis and damage and to perform stem cell functions. This study investigated changes occurring in neurons and glia in the central nervous system following metamorphosis in axolotls. Morphological alterations in brain tissue, newly formed neurons, and cellular organizations in different brain regions were assessed using stereological and immunohistochemical methods, as well as light and electron microscopy. Interestingly, we observe no statistically significant difference in total neuron numbers in the telencephalon region between neotenic and metamorphic axolotls. However, the proliferation index and the numbers of cells expressing NeuN were significantly higher in metamorphic axolotls. Furthermore, structural changes in neuronal nuclei and myelin sheath organization were determined at the light and electron microscopic levels post‐metamorphosis. Ultrastructural analyses revealed a change in chromatin organization from euchromatic to heterochromatic in neurons after metamorphosis, and morphological changes were also demonstrated in myelinated nerve fibers in the telencephalon. Additionally, mucopolysaccharide‐containing secretory sacs were also identified on the apical surfaces of a subgroup of ependymoglia cells located in the lateral ventricle wall. Overall, this study sheds useful light on the intricate changes occurring in the central nervous system during metamorphosis in axolotls and provides valuable insights into the mechanisms underlying these processes.

## Graphical abstract

In the telencephalon, apical specializations such as cilia, primary cilia, and microvilli with varying morphologies were observed. Goblet‐like cells, arranged in a single row along the ventricular wall, contained secretory vesicles in their apical regions. Light microscopy demonstrated that these secretory vesicles had a carbohydrate‐rich structure, as confirmed by positive alcian blue staining. Ultrastructural analysis revealed detailed cellular features within the ventricular wall. After metamorphosis, changes in the chromatin structure of the cell nucleus and alterations in the myelin sheath structure were observed.

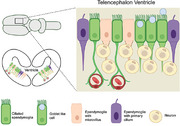

## 1 Introduction

The axolotl (*Ambystoma mexicanum*) is an aquatic salamander known for its remarkable ability to regenerate various body parts, such as the brain, limbs, internal organs, and heart (Yun 2021).

In the majority of salamander life cycles, individuals undergo metamorphosis, thus transitioning into fully developed adults. However, the axolotl deviates from this norm. This organism retains its larval characteristics throughout its life, displaying neoteny and permitting unlimited growth. Newts possess a developmentally regulated switch from stem cells to dedifferentiation for limb muscle regeneration (Suetsugu‐Maki et al. 2012; Tanaka et al. 2016; Yun 2021). Alternatively, axolotls adjust to terrestrial living conditions by introducing thyroid hormone (TH) into their habitat. Instances of organ or extremity loss or reconstruction, as well as the formation of new organs, may occur throughout this adaptive process (Demircan et al. 2018).

Although brain development follows an evolutionarily conserved pattern among vertebrates, brain anatomy varies significantly among species. The developmental processes involve the generation of new neurons, cell proliferation, and cell cycle arrest (Joven and Simon 2018). The salamander brain contains cells known as ependymoglia in the ventricular region. These ependymoglia cells demonstrate proliferative properties in response to disruptions in homeostasis or damage (Amamoto et al. 2016; Lust and Tanaka 2019). These progenitor cells play a role during development in different salamander species. Additionally, studies focusing on brain development in salamanders undergoing a metamorphosis in their life cycle have shown that ependymoglia cells differentiate and contribute to the formation of new neurons (Lust and Tanaka 2019). Similarly to glial cells, salamander ependymoglia cells also possess neurogenic potential and play important roles in injury‐induced neurogenesis in the adult brain (Berg et al. 2011; Kirkham et al. 2014). Previous studies have demonstrated molecularly that there are subgroups of ependymoglia cells lining the axolotl ventricle wall (Lust et al. 2022).

Its extraordinary regenerative capacity has positioned the axolotl as a prominent organism for investigating the mechanisms underlying tissue regeneration. Experimental animals such as the axolotl, with their exceptional regenerative powers and distinctive features, play a crucial role in advancing our understanding of the natural world. They contribute significantly to the development of innovative therapeutic and preventive strategies for enhancing human health.

The aim of the study is to investigate the effects of inducing metamorphosis on the axolotl brain and analyze the ultrastructure of neurons and glia cells.

## 2 Methods

### 2.1 Ethics and Animal Care

The study commenced following receipt of approval from the Istanbul Medipol University animal experiments local ethics committee (number E‐38828770‐772.02‐49005). Neotenic and metamorphosis‐induced axolotls (*A. mexicanum*, RRID:AGSC_100A), all aged 12 weeks, obtained from the Istanbul Medipol University Medical Research Center, were used in the study. The animals were housed individually in an aquarium and maintained in Holtfreter's solution at a temperature of 18 ± 2°C, following the protocol outlined in a prior study, throughout the duration of the experiments (Demircan et al. 2019). Metamorphosis was induced according to the protocols described by Bay et al. (2023). Tetraiodothyronine (T4 solution, 50 nM) solution was prepared by dissolving thyroxine in Holtfreter solution, and each axolotl was kept in these solutions in a separate container (Bay et al. 2023; Demircan et al. 2021). Progression to the metamorphic stage was carefully monitored by observing morphological changes (weight loss and loss of fins and gills). Following complete metamorphosis, the animals were kept without hormone treatment for a month to allow them to adapt to terrestrial living conditions. Power analysis was performed with Minitab software (version 20). The appropriate number of animals for the stereological analysis was determined to be five, and five animals were therefore included in each group for the stereological analysis.

### 2.2 Histology and Stereology

The brain tissues were fixed in a 1:1 ratio of 4% paraformaldehyde (PFA) + 4% glutaraldehyde solution for 1 week at +4°C and subjected to paraffin tissue processing. A stereological technique, the physical disector counting method, was applied for cell counting. Disector pairs of 4 µm‐thick sections were taken from paraffin blocks at a sampling rate of 1:24. After deparaffinization, these were stained with 1% Cresyl violet.

Power analysis was carried out using Minitab software (version 20), which showed that the number of animals used for the analysis test should be five. The total number of cells in the telencephalon region was estimated by physical fractionation. The systematic random sampling method was applied to the sections, and photographs were captured using a light microscope (Olympus Tokyo, Japan) with cellSens software (Olympus SC50, Olympus Soft Photographed using Imaging Solutions GmbH, Germany). Coefficient of error (CE) and coefficient of variation (CV) values were calculated based on the counting results for the groups and individual animals (Gundersen and Jensen 1987). Alcian blue stainings (Bio Optica, 04‐160802) were performed according to the kit protocols for determining the content of ependymoglia's secretory product.

### 2.3 Immunohistochemical Analyses

The systematic random sampling method was applied to paraffin sections 4 µm in thickness. They were sampled at a ratio of 1:180 and mounted onto positively charged slides. Immunohistochemical analyses were conducted following the kit protocol (Mouse Rabbit specific HRP/AEC ABC Detection Kit, ab93705, Abcam). The antigen retrieval process was carried out with heat‐induced epitope retrieval citrate buffer (10X) (AP‐9003‐125, Thermo Scientific) diluted in PBS (final concentration is 1X) for 2.5 min at 800 Watts and 12 min at 180 Watts by using the microwave. Primary antibodies (Anti‐Myelin Basic Protein (1:50, ab40390, Abcam, RRID:AB_1141521), Anti‐Ki‐67 (1:500, ab15580, Abcam, RRID:AB_443209), Anti‐NeuN (1:500, MAB377, Chemicon, RRID:AB_2298772), Anti‐Glial Fibrillary Acidic Protein (1:300, MAB360, Chemicon, RRID:AB_11212597) were used by dilution with PBS. Counterstaining was carried out with Mayer's hematoxylin (51275, Sigma‐Aldrich). Images were captured from the sections through systematic random sampling and were processed using ImageJ software (National Institutes of Health). A superimposed point counting grid in ImageJ software, 500 µm^2^ per point, was placed over the photographs, and the positive cell volume was calculated by using the Cavaliere method.

### 2.4 Electron Microscopy

#### 2.4.1 Transmission Electron Microscopy

The fixed tissues were postfixed with osmium tetroxide (OsO_4_) (1%) for 1 h. Next, they were washed four times for 15 min each with 0.1 M PBS, ensuring thorough washing throughout. Dehydration was carried out by passage through an increasing acetone series. The specimens subsequently underwent a propylene oxide step, with two 20‐min cycles and final preservation in 100% pure propylene oxide. The embedding material was prepared according to the Araldite CY212 kit protocol. Semi‐thin sections (500 nm) were taken from the resin blocks using a glass knife, and thin sections (70 nm) were collected using a diamond knife with an ultramicrotome (EM UC7, Leica). The semi‐thin sections were stained with 1% toluidine blue and examined under a light microscope (Bancroft and Gamble 2008). Thin sections, mounted on 200‐mesh grids, underwent contrasting using a 0.05% uranyl acetate and 2% lead citrate solution with a contrasting device (EM AC20, Leica).

The second tissue preparation protocol was adapted from Yi̇ği̇t et al. (2023) for the detailed analysis of neotenic axolotl ependymoglias. In this method, tissues were sampled using a brain matrix and fixed with Karnovsky's fixative for 3 days. Subsequently, the tissues underwent postfixation in an aqueous solution of OsO_4_ (2% OsO_4_ + 1.5% potassium ferrocyanide) for 30 min at room temperature. Following this step, incubation with 0.5% thiocarbohydrazide for 30 min and 2% OsO_4_ for 30 min was carried out at room temperature. In order to enhance contrast, tissues were treated with 2% uranyl acetate overnight and with lead aspartate for 50 min. After each incubation step, the tissues were thoroughly washed three times with ddH_2_O. Following the washing steps, the tissues were subjected to a sequential series of graded acetone solutions, including concentrations of 50%, 70%, and 90%, and two rounds of 100%. Subsequently, the tissues were incubated in acetone‐epoxy resin mixtures and finally embedded in 100% epoxy resin (45359, Sigma‐Aldrich). Electron microscopic analyses were conducted on images captured at different magnifications using JEOL JSM‐7001F and Zeiss Gemini 500.

#### 2.4.2 Scanning Electron Microscopy

The brain tissues were fixed using a solution of 4% PFA + 4% glutaraldehyde at +4°C. They were then sliced into 1‐mm sections using a brain matrix. Tissues were washed with PBS, and postfixation was carried out using 1% OsO_4_. After rinsing with distilled water, the tissues underwent a 15‐min series of acetone dehydration (50%, 70%, 90%, and 100%). Supercritical drying was performed using a Leica CPD300 device. A gold‐palladium coating was applied for 30 s using a Leica EM ACE200 coating device with a diffuse setting. Imaging was performed at various magnifications using a secondary electron detector and a 3 kV accelerating voltage on the Zeiss Gemini 500 electron microscope.

### 2.5 Statistical Analysis

Statistical analyses were conducted on GraphPad Prism 9.0.0 software (GraphPad Software, LLC, RRID:SCR_002798). Comparisons between the groups were carried out using the Shapiro–Wilk test and Student's *t*‐test. Statistical significance was set at *p* ≤ 0.05.

## 3 Results

### 3.1 The Total Number of Neurons in the Telencephalon Remained Unchanged Following Metamorphosis

**FIGURE 1:**
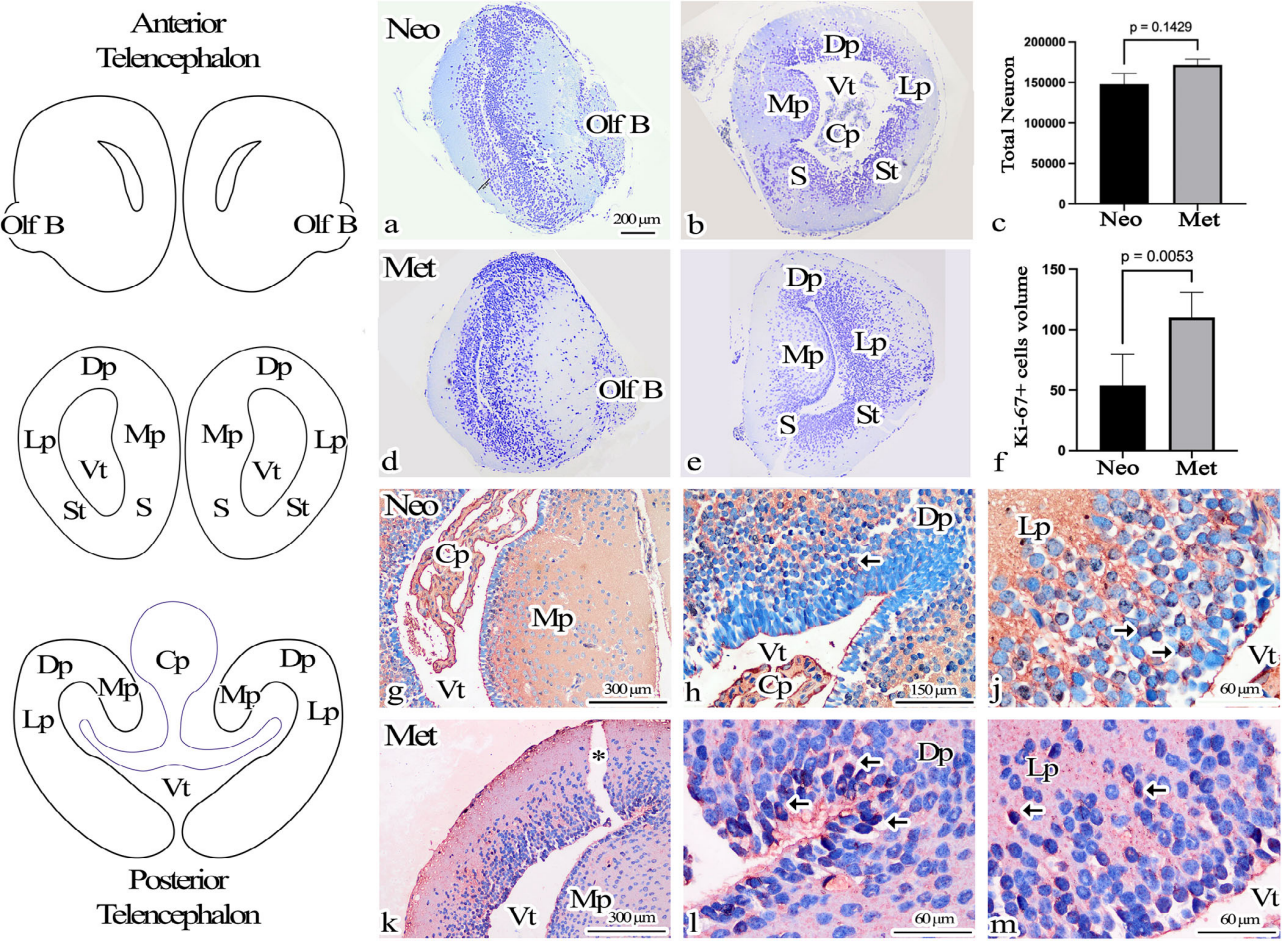
(a,b,d,e) Cresyl violet staining brains of neo and met axolotls. (c) Physical fractionator cell count results. (f) Total cell volume results of Ki‐67 positive cells. (g–m) Ki‐67 immunohistochemistry staining of the telencephalon regions of neo and met axolotl brains. There are Ki‐67‐expressing neurons (arrow) at different distances from the ventricle. (l, m) Magnifications of (k). Arrow: Ki‐67 positive cell, *: artifact, Cp: choroid plexus, Dp: dorsal pallium, Lp: lateral pallium, Mp: medial pallium, Olf B: olfactory bulb, S: septum, S: striatumVt: ventricle.

For light microscopic examination and estimation of total neuron numbers. The total numbers of neurons obtained from the sections analyzed are shown in Table 1 and Figure 1a–e. CE and CV values were calculated based on the counting results, and both were within the confidence range. Statistical analysis revealed no significant difference between the groups in terms of total neuron numbers; *p* = 0.14 (shown in Figure 1c).

**TABLE 1:** Physical fractionator analysis results.

Group	CV	CE (mean)	*SD*	Total cell number (mean)
Neo	0.14	0.04	23,328	146,842
Met	0.08	0.05	14,724	171,418

### 3.2 Metamorphosis Maybe Associated With a Rise in Both the Proliferation Index and the Population of Mature Neurons

Immunohistochemical analysis was performed to collect information regarding the status of cells following metamorphosis. Ki‐67‐expressing cells were identified at varying distances from the ventricle in the telencephalon (shown in Figure 1g–m). A comparison of the total volumes of Ki‐67‐expressing cells between Neo and Met group brains revealed a statistically significant increase in the metamorphic group; *p* = 0.005 (shown in Figure 1f).

**FIGURE 2:**
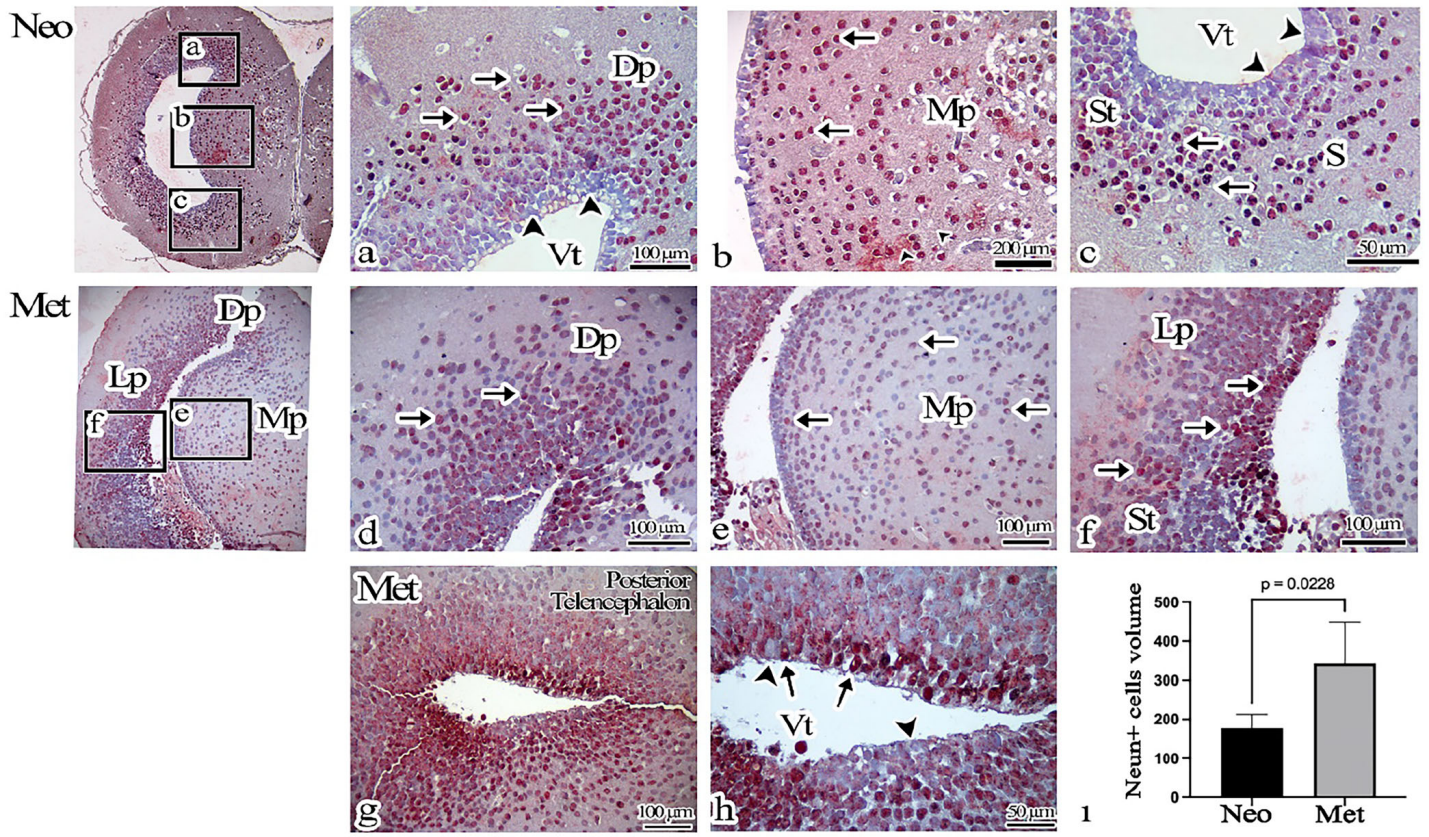
(a–h) NeuN immunohistochemistry staining of the telencephalon region of the Neo and Met axolotl brain. NeuN‐expressing cells (arrow) are located at different distances from the ventricular wall. (ı) Total cell volume results of NeuN positive cells. Arrow: NeuN positive cell, arrowhead: NeuN negative cell. Dp: Dorsal pallium, Lp: lateral pallium, Mp: medial pallium, S: Septum, S: Striatum, Vt: ventricle.

Cells expressing NeuN were observed at various distances from the ventricular wall in both groups (shown in Figure 2a–f). However, in the Met group, NeuN‐expressing cells were specifically detected in regions where ependymoglia cells lined the ventricular wall (shown in Figure 2g,h). A comparative analysis between the two groups was conducted based on NeuN‐expressing cells in the same samples. A comparison of the total volumes of NeuN‐expressing cells in Neo and Met group brains revealed a statistically significant increase in the Met group (*p* = 0.0228) (shown in Figure 2l).

**FIGURE 3:**
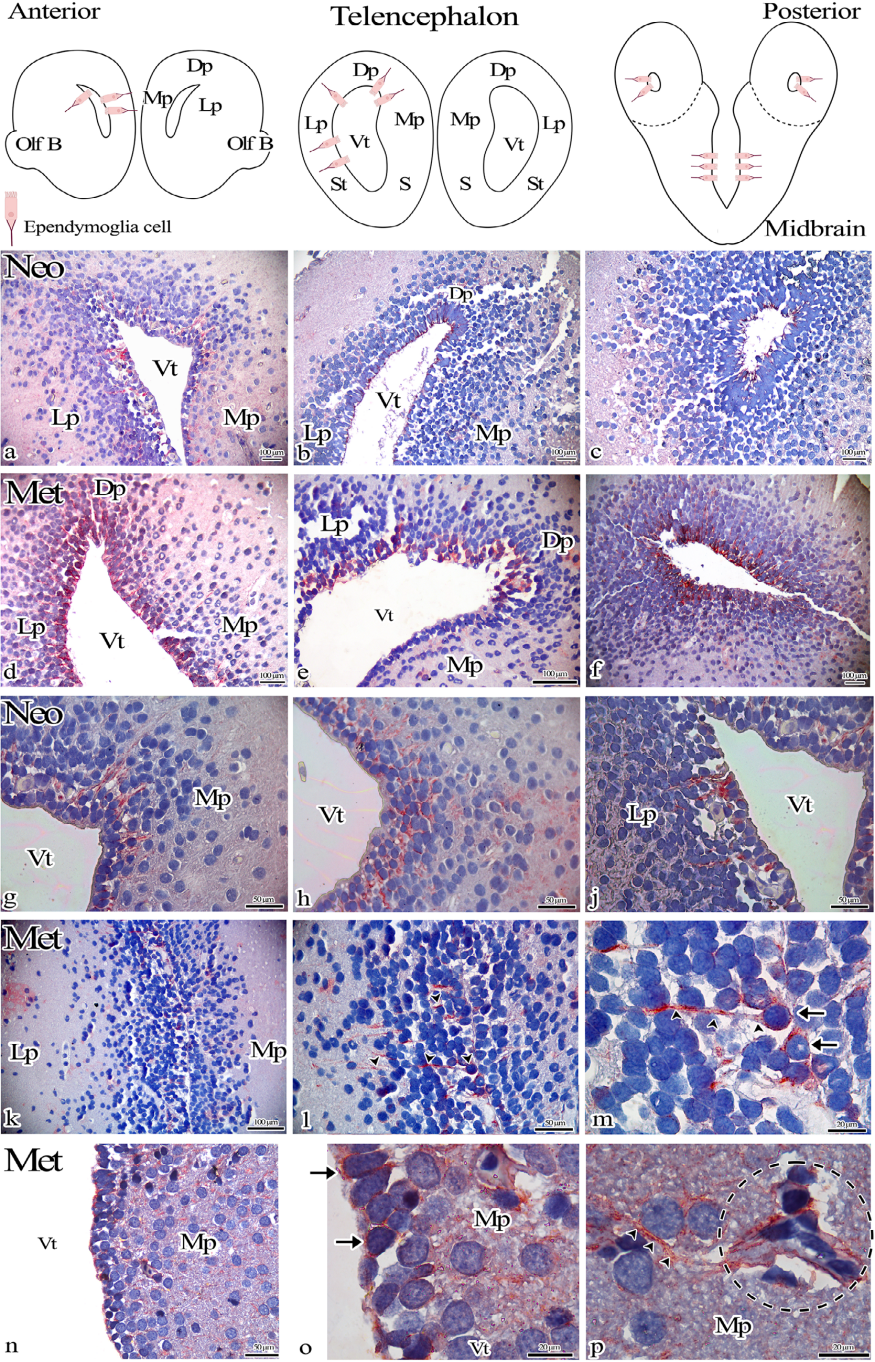
Schematic view of anterior, midpiece, and posterior portions of the telencephalon. (a–p) GFAP immunohistochemistry staining of the telencephalon regions of Neo and Met axolotl brains. GFAP‐expressing cells (arrow) and their extensions (arrowhead) located in the ventricular wall are shown. (m) Magnifications of (l). (p) GFAP‐expressing cell extensions can be seen surrounding the blood vessel. Dp: Dorsal pallium, Lp: lateral pallium, Mp: medial pallium, S: septum, S: striatum, Vt: ventricle.

GFAP‐expressing cells were examined in sections from the anterior (shown in Figure 3a,d) and posterior (shown in Figure 3c,f) telencephalon regions from the Neo and Met group axolotl brains (shown in Figure 3a–j). Analyses of the telencephalon region showed that ependymoglia cells expressed GFAP in axolotls in both groups. However, the extensions of GFAP‐expressing cells were observed to extend to larger areas in the Met group (shown in Figure 3k–p).

**FIGURE 4:**
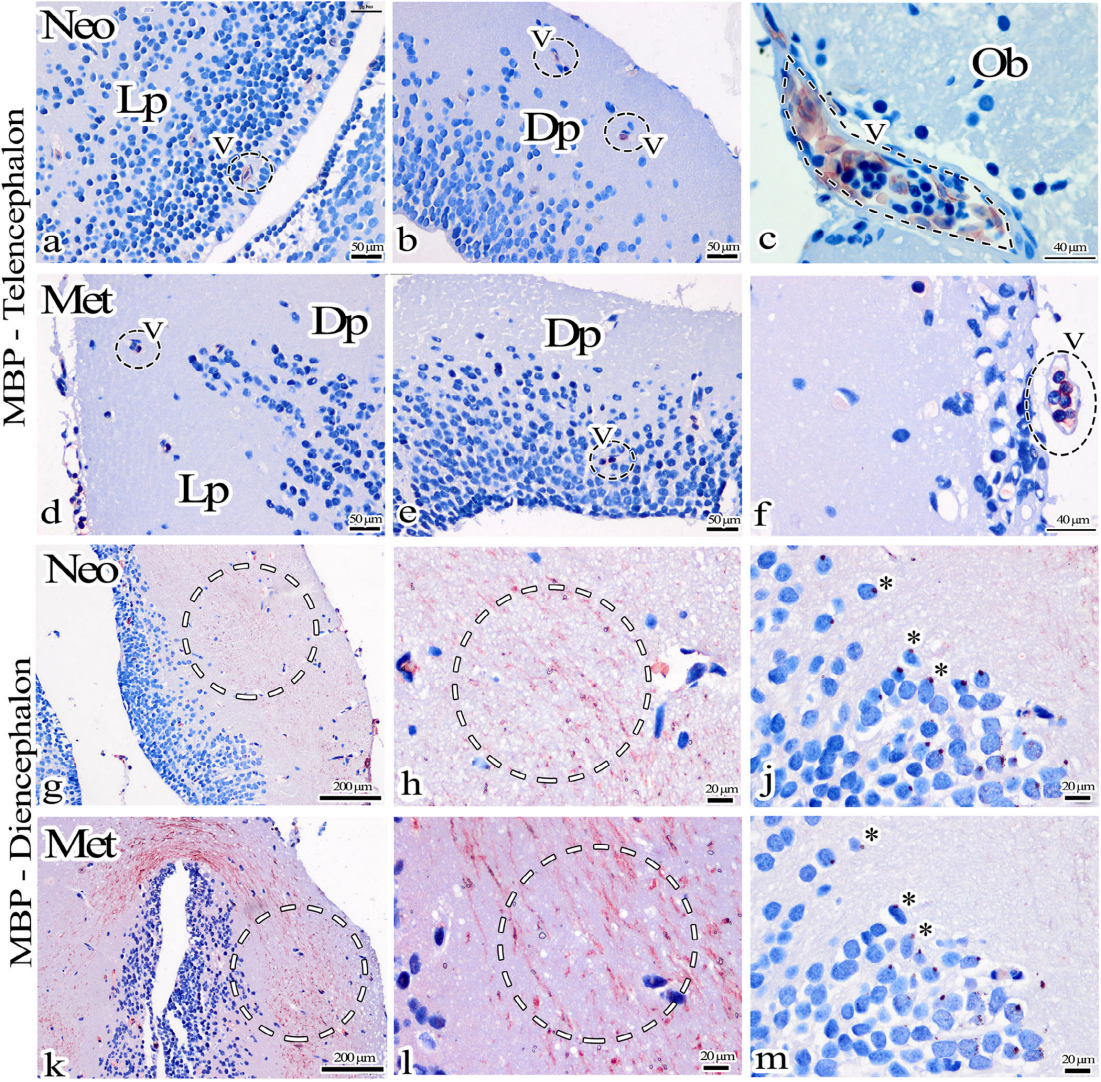
(a–f) MBP immunohistochemistry staining of the telencephalon region of the Neo and Met axolotl brain. MBP‐expressing nerve fibers could not be detected in the telencephalon. However, blood cells (dashed circles) were found to express MBP. (g, h, k, l) MBP immunohistochemistry staining of diencephalon regions of Neo and Met axolotl brains. MBP‐expressing nerve fibers are seen in the dashed circle. (j, m) When serial twin sections taken from the diencephalon region are examined, cells containing red granules (*) organized in groups are seen. Dp: dorsal pallium, Lp: lateral pallium, Ob: olfactory bulb, V: blood vessel.

Both groups of axolotls expressed myelin basic protein (MBP) in red blood cells in the telencephalon region, while glial cells exhibited no MBP expression (shown in Figure 4a–f). Statistical analyses could not be performed in the telencephalon due to this absence of MBP expression in glial cells and myelinated nerves. When rat brain tissue was utilized as a positive control, indicating MBP expression, myelinated nerve fibers in the hippocampus were found to be positively marked (shown in Figure S1). In the diencephalon, MBP immunohistochemistry analysis revealed regions expressing MBP in both the Neo and Met group axolotls. Notably, increased MBP expression was observed in the Met group axolotls (shown in Figure 4g,h,k,l).

### 3.3 Metamorphosis May Trigger the Onset of the Demyelination Process

**FIGURE 5:**
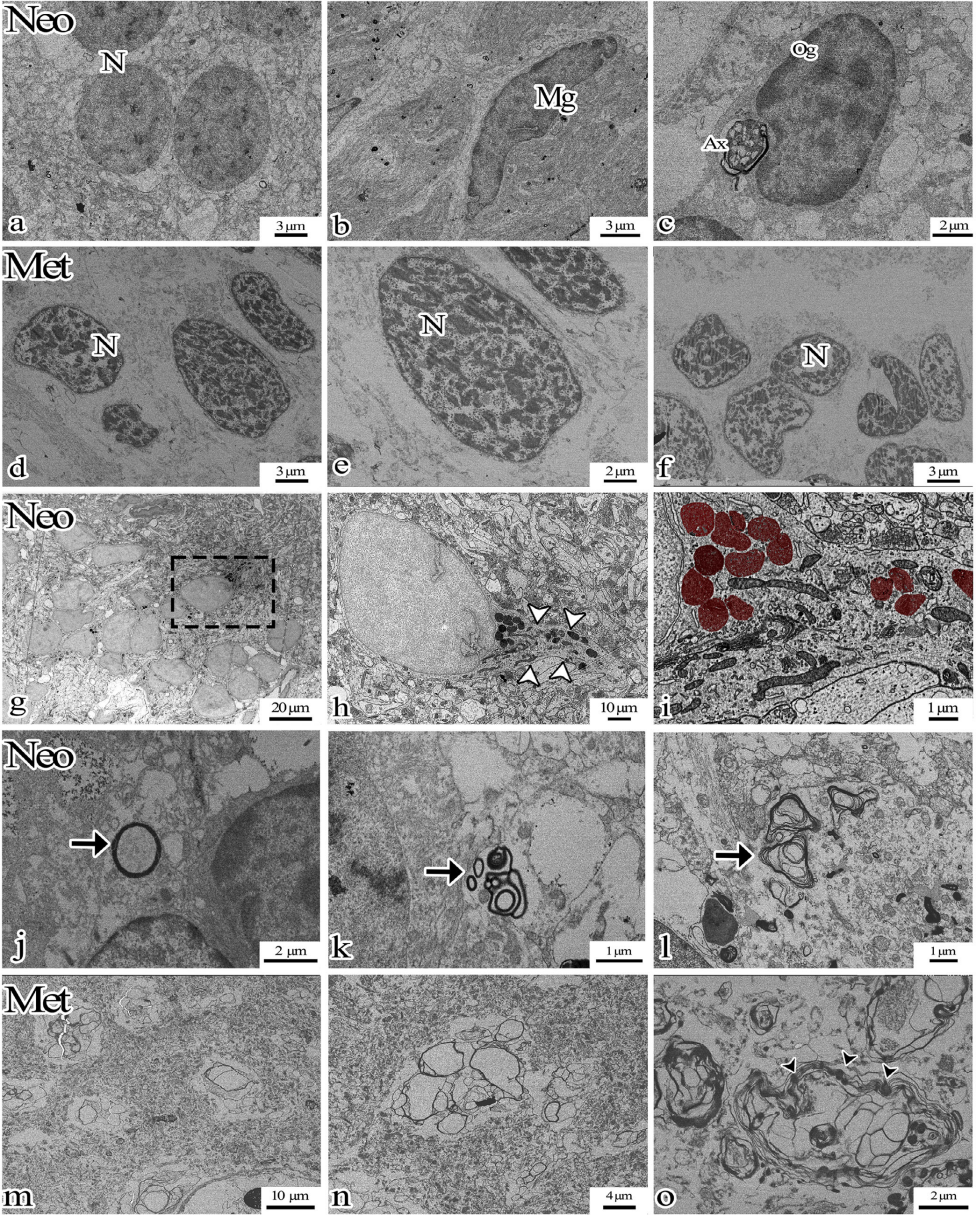
(a) Neo neurons (N) are seen. (b) A microglia‐like cell (Mg) surrounded by neuropil (c) oligodendroglia (Og) producing a myelin sheath is seen. (d–f) Morphologically changed neurons (N) are seen in the metamorphic axolotl. (g–i) Transmission electron microscopic images taken from the diencephalon region of the neotenic axolotl brain. (h) Black granular structures in the regions of group‐organized neurons facing the pial surface. (i) The structures marked in red in the cell cytoplasm maybe neuromelanin. (j–o) Transmission electron microscopic images taken from the telencephalon region of the neotenic axolotl brain. A nerve fiber (Ax) with a compact (j, k, m, n) and non‐compact (l, o) myelin sheath is examined (white arrowhead: soma, black arrowhead: impaired myelin structure, arrow: myelinated nerve; the dashed rectangle in g is magnified in h and i).

The study findings revealed distinct ultrastructural morphological features between Neo and Met group axolotls (shown in Figure 5). Electron microscopic analyses revealed myelinated nerve fibers of varying sizes and diameters in the anterior and posterior telencephalon (shown in Figure 5). Myelinated nerve fibers in the Met group axolotl displaying differing myelin lamellar structures were identified in the telencephalon region (shown in Figure 5j–o).

### 3.4 The Euchromatic Nucleus Undergoes Reorganization Into Heterochromatin During Metamorphosis

Examination of the Met group axolotl telencephalon revealed the marked presence of vesicular spaces in the neuronal nuclei. Both Neo and Met group axolotl neuronal nuclei occupied a significant proportion of the cytoplasm. However, distinctions were noted between the two groups, with Neo group neurons exhibiting a euchromatin structure in their nuclei, while Met group neurons displayed a heterochromatic structure (shown in Figure 5a–f). Additionally, microglia‐like cells and oligodendroglia were observed in the Neo group (shown in Figure 5b–c).

### 3.5 Cells Containing Neuromelanin Were Observed in the Axolotl Brain

MBP immunohistochemical labeling revealed clusters of cells containing MBP‐positive or AEC chromogen‐positive granules in the diencephalon serial twin section (shown in Figure 4j,m). Electron microscopic examination was performed in order to obtain a better understanding of these cells. This revealed electron‐dense granules within the cell cytoplasm (shown in Figure 5g–i). Examination of deparaffinized paraffin sections with no staining revealed dark‐colored granules (shown in Figure S2). Light and electron microscopic analyses in the diencephalon suggest that these granules maybe cellular neuromelanin.

### 3.6 Goblet‐Like Cells Were Identified in the Telencephalon

**FIGURE 6:**
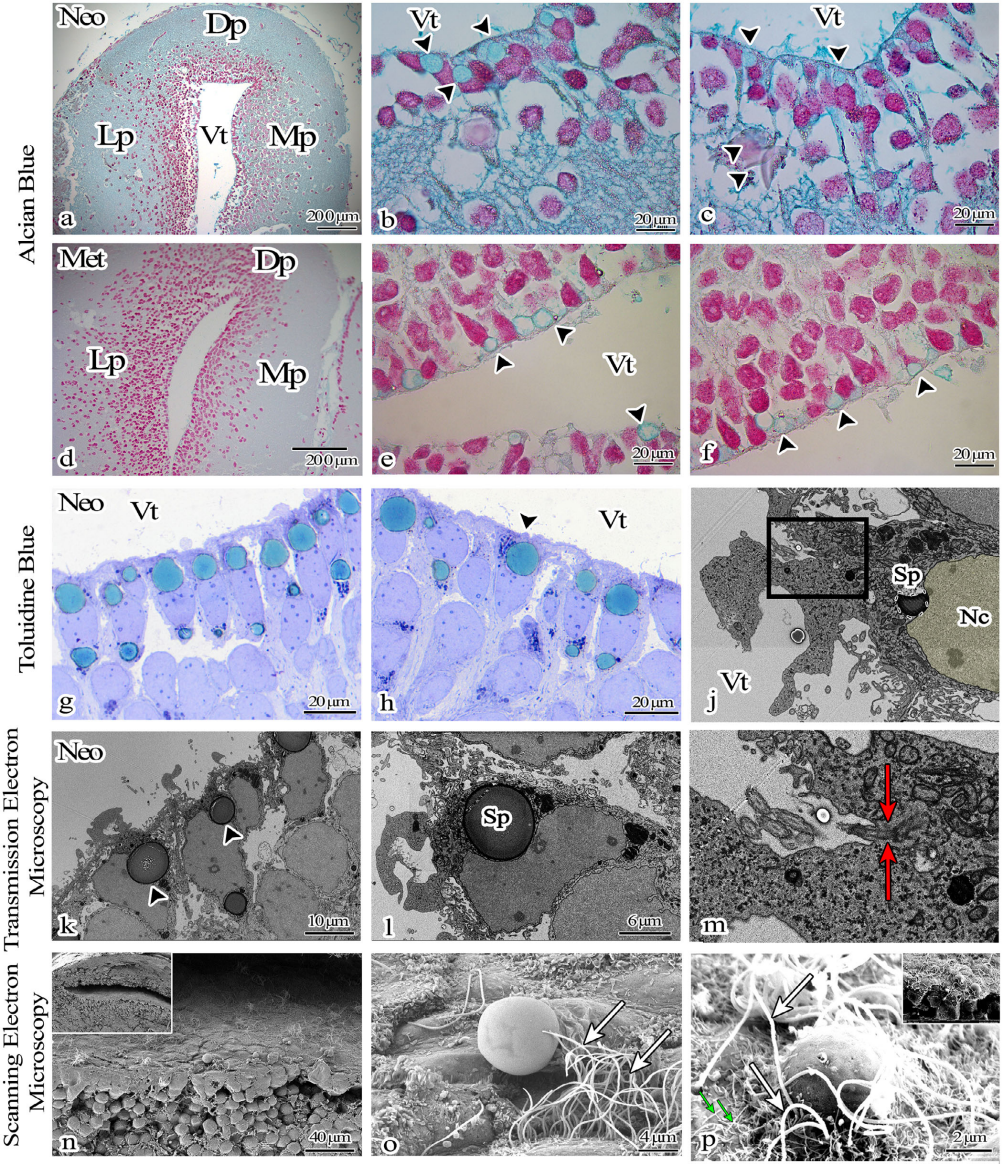
Light (a–h), transmission (j–m), and scanning (n–p) electron microscopic images of the secretion product (arrowhead, Sp) located in the apical part of the ependymoglia cells arranged around the ventricle in the telencephalon region of the Neo and Met axolotl brain. The crystalloid structure of the secretory granule was seen. (a–f) Sacs were stained with alcian blue (arrowhead). (g, h) Semi‐thin section of ependymoglia cells and sacs within different cells shown with arrowhead. (j, k, l) Secretory products (arrowhead, Sp) at the apical surface of ependymoglia cells were seen. (m) At higher magnification of j, red arrows showed the basal body of the cilium of the cell (o). Higher magnification of n, secretion product on the ventricle wall was seen, and the cilium of the cell was shown with a white arrow. (p) Secretion products protruding towards the ventricle are seen on the apical surfaces of some ependymoglia cells; cilia (white arrow) and microvilli (green arrow) are located at the apical surface. Nc: nucleus, Sp: secretion product, Vt: ventricle.

Secretory sacs were identified in the apical part of prismatic‐shaped ependymoglia cells, arranged in a single row on the ventricle wall in both Neo and Met group axolotl brains (shown in Figure 6a–h). During the classic electron tissue processing procedure, the contents of the sacs located on the apical surface of these cells were not preserved with OsO_4_ (shown in Figure S3). Since they could not be fixed with osmium fixation, we speculated that they might be carbohydrate‐derived. Electron microscopic analysis, while inconclusive in terms of the precise content of the secretory vesicles, suggested a carbohydrate‐rich nature, corroborated by positive staining with alcian blue at light microscopic examinations (shown in Figure 6a–f). The ultrastructure of these vesicles resembled the secretory vesicles typically found in mammalian goblet cells (shown in Figure S3). A new electron tissue processing method (en‐bloc staining) involving different fixation methods was employed. The contents of these sacs on the apical surface were preserved with this method (shown in Figure 6k,l).

**FIGURE 7:**
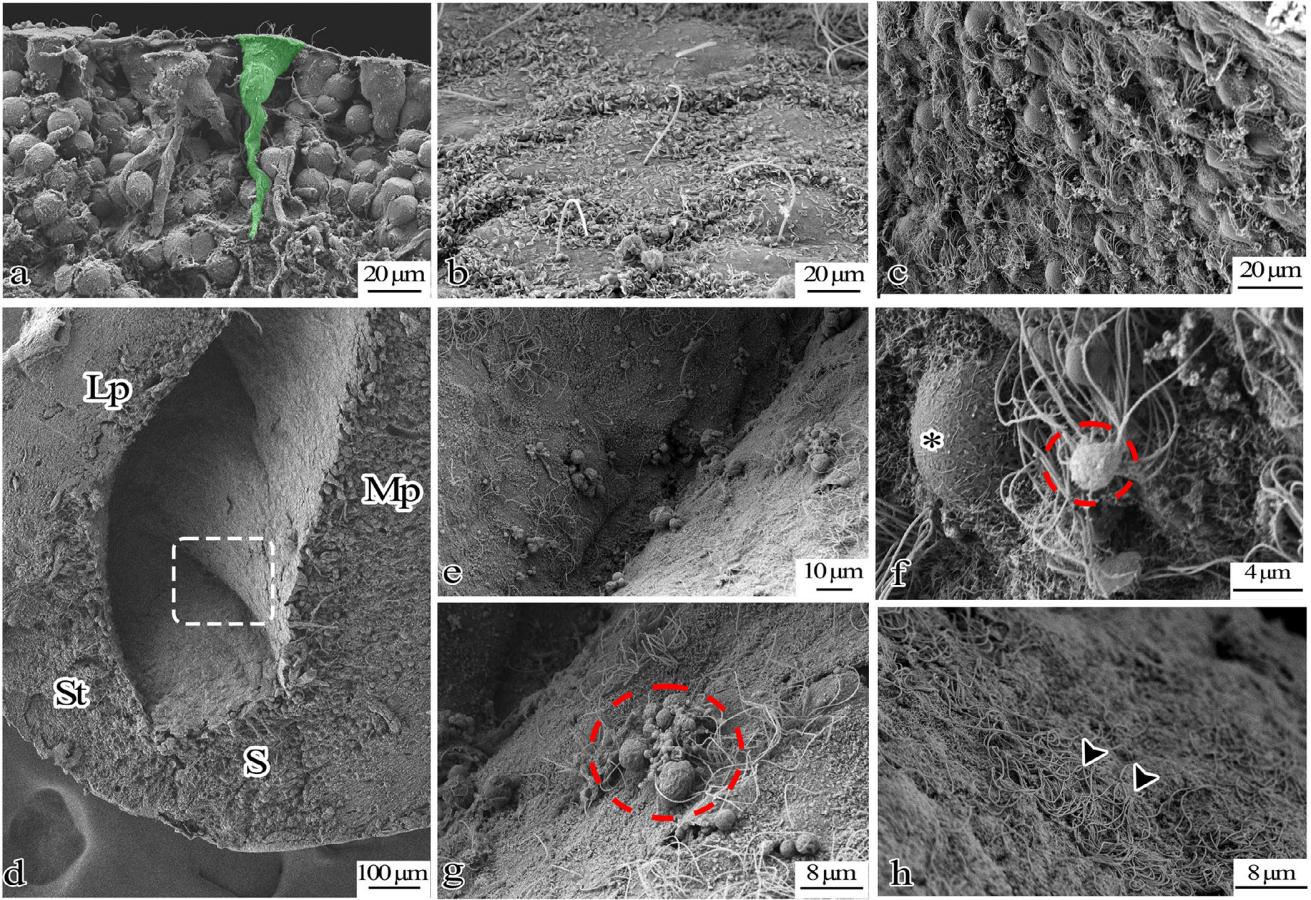
Scanning electron microscope images of the telencephalon of the neotenic axolotl brain. (a) Ependymoglia cells with long cytoplasmic extensions are seen (green). (b) Ependymoglia cells with primary cilium are seen. (d) Ventral telencephalon. (e, g) higher magnification white dashed rectangular in d. (c, f, h) There are many apical surface specializations (arrowheads) on the apical parts of the cells on the surface of the wall facing the ventricle; some cells do not have any cilia (*) (g, f) A structure that maybe a secretory sac is seen between the cilia (red dashed circle).

In the Neo group axolotl brain critical‐dried for scanning electron microscope analyses, prismatic‐shaped ependymoglia cells were tightly arranged around the ventricle, their apical surfaces were covered with a distinct layer comprised of numerous cilia (shown in Figure S4), and some ependymoglia had long cytoplasmic extensions (shown in Figure 7a). Ciliary structures with varying morphologies and cells organized in a pattern resembling the olfactory epithelium were observed in the ventricle wall of the telencephalon (shown in Figure 7c,e–g) and hypothalamus (shown in Figure S5 a‐d) of the diencephalon. While some cells lacked distinctive structures on their apical surface and they have microvilli or primary cilia, those in another region displayed cilia structures on the apical surface (shown in Figures 6n–p and 7). Secretory sacs were discharged from the apical surface of the cells in the ventricular wall, and numerous cilia around the sacs were detected in the telencephalon (shown in Figures 6k–p and 7).

## 4 Discussion

This study explores the benefits of providing crucial data concerning the reorganization and reconstruction of histological events within axolotl brain tissue, thus contributing to the field of neuroscience. Neotenic axolotl brain induced to undergo metamorphosis was subjected to morphological characterization and segmentation.

The power analysis test established that each group should consist of five animals. The total number of cells in the telencephalon region of the axolotl was determined using the physical disector counting method, a stereological technique. CE and CV values were calculated based on the counting results obtained (Gundersen and Jensen 1987). The consistency of these values within the confidence interval confirmed the homogeneity of distribution within the groups and validated the accuracy of the sampling strategy. We think that five axolotls maybe sufficient for future studies involving the examination of neurons in the telencephalon region using stereological methods.

Brain development in humans and many mammalian species involves a complex process that continues until adulthood (Castillo‐Ruiz et al. 2020). This developmental process comprises a sequence of events involving the formation of new neurons and the death of cells. Developmental cell death is a critical aspect of brain maturation in vertebrates. During the perinatal period, approximately half the neurons initially generated in mammals undergo apoptosis. Developmental neuronal cell death is characterized by the activation of caspase‐3 and subsequent apoptosis, leading to the demise of intracellular proteins (Castillo‐Ruiz et al. 2020). Ahern et al. (2013) revealed that the peak period of cell death in mice predominantly occurs within the first week after birth; the highest level is observed in the initial postnatal 3 days across various brain regions. The cell demise associated with birth maybe influenced by various hormones and pro‐inflammatory cytokines (Castillo‐Ruiz et al. 2020).

Immunohistochemical analyses employing an anti‐Ki‐67 proliferation marker on sections taken from the telencephalon region via systematic random sampling revealed a significant increase in the numbers of cells expressing Ki‐67 in the Met group relative to the Neo group axolotls. This increased number of Ki‐67‐expressing cells in the Met group axolotls suggests active neuronal proliferation. The T4 hormone administered to facilitate metamorphosis in the Neo group axolotls may have stimulated the proliferation of cells within the telencephalon region (Kress et al. 2009).

Our stereological analyses also revealed that the estimated numbers of neurons in the telencephalon region remained unchanged during metamorphosis. Although no statistically significant alteration was detected in the total numbers of telencephalic neurons post‐metamorphosis, it is intriguing that a statistically significant rise occurred in the number of cells expressing Ki‐67, a marker for proliferation. NeuN immunohistochemical analysis was performed in order to identify mature neurons in the telencephalon regions in the Neo and Met group axolotl brains, which revealed a significant increase in the number of mature neurons in the Met group compared to the Neo group. This increase in the number of maturing cells may account for the lack of any difference in total cell numbers between the two groups according to our stereological analysis results comparing total cell counts. The discrepancy between the high number of proliferating neurons and the unchanged total cell count might also be attributable to rapid cell death during metamorphosis. Apoptosis observed during amphibian metamorphosis is triggered by a single hormone, TH (Ishizuya‐Oka et al. 2010). Briefly, metamorphosis may have triggered a process akin to the neuronal cell death observed in mammals during the perinatal period. During amphibian metamorphosis, a significant reorganization of the central nervous system occurs as the organism transitions from the aquatic larval stage to the terrestrial adult stage, necessitating adaptations in behavior and morphology (Coen et al. 2007; Ishizuya‐Oka et al. 2010). For example, in *Xenopus laevis*, apoptosis plays a critical role in this reorganization process. Although not directly observed by EM or TUNEL analysis, caspase‐9, an important apoptotic factor, is active during NF Stages 55–63, with peak activity at Stage 60. Its mRNA is expressed in the ventricular zone (VZ), a neurogenic region of the brain (Ishizuya‐Oka et al. 2010). Apoptotic cells are localized to specific regions, including the ventral forebrain and subpallium, with the elimination of Mauthner neurons and other larval sensory and motor neurons (Ishizuya‐Oka et al. 2010). Brain remodeling during metamorphosis balances apoptosis, proliferation, and differentiation mediated by mitochondrial death pathway components such as caspase‐9 and XR11 (Coen et al. 2007). These findings highlight the essential role of apoptosis in amphibian brain remodeling, suggesting that similar mechanisms may contribute to structural and functional changes in other vertebrates. Further investigations employing apoptosis markers are now needed to ascertain the direction and mechanisms underlying this process. Additionally, such studies will help elucidate the organization and rates of apoptotic cells in the medial, lateral, and dorsal telencephalon throughout various stages of metamorphosis.

The role of ependymal cells in urodel spinal cord regeneration has been extensively explored to date. Research indicates that these cells participate not only in growth factor and retinoid responses and extracellular matrix dynamics but also in cytoskeletal alterations, axonal growth, exhibit stem cell functions, and contribute to the restructuring of the dorsal–ventral network (Enos et al. 2019). Studies of adult zebrafish brain tissue have identified various proliferation zones in which neurons are continuously generated (Adolf et al. 2006; Chapouton et al. 2007). These and other findings suggest that the widespread distribution of homeostatic neurogenesis in the brain maybe attributed to an underlying component responsible for the extensive regenerative capacity observed in these animals (Kaslin et al. 2008). However, this hypothesis requires further investigation, as it raises the possibility of mobilizing non‐germinal regions for functional neuronal replacement in species in which such natural occurrence is not observed (Berg et al. 2010).

Newt and axolotl studies have revealed that these cells that line the ventricular wall of the telencephalon are involved in brain development, growth, and regeneration and also represent the source of new neurons. The fact that these cells express glial fibrillary acidic protein (GFAP), glutamine synthase, and aquaporin 4, markers of astrocytes, suggests that they possess similar properties to those of radial glial cells, progenitor cells in the mammalian nervous system (Becker and Becker 2015). These cells found in adult amphibians and fish are therefore known as ependymo‐radial glia (ERG) (Reimer et al. 2008). ERGs have also been described as “radial glia” (Fei et al. 2014), “tanycytes” (Reichenbach and Wolburg 2012), or “ependymoglia” (Kirkham et al. 2014). Lust et al. (2022) identified 15 different transcriptional ERG cell groups. These cells are classified under three main groups, “quiescent ependymoglia,” “active ependymoglia,” and neuron precursor “novel ependymoglia.” In the developing mammalian neocortex, neuronal progenitors can be categorized based on their mitotic location: apical progenitors (APs) divide at the apical surface of the VZ, while basal progenitors (BPs) carry out mitosis in the subventricular zone (SVZ). The long basal processes of BPs have been described to play an important role in transferring self‐renewal capacity to daughter progenitors and facilitating the migration of upper‐layer neurons. When BPs differentiate into astrocytes, their morphology changes (Nomura et al. 2016). Mammal‐like astroglia have not been observed in either anurans or urodeles. In mammals, radial glial cells are thought to play a critical role in guiding neurons to their designated locations during migration (Naujoks‐Manteuffel and Roth 1989).

In our study, the telencephalon region of axolotls and ependymoglia cells was found to express GFAP in both groups. However, in the Met group, extensions of GFAP‐positive cells were seen to extend towards the pial surface. Studies involving the brains and spinal cords of amphibians and fish have shown the presence of cells arranged around the ventricle that extend the footpad toward the pial surface and that these cells represent the source of new neurons (Becker and Becker 2015; Berg et al. 2011; Dirian et al. 2014). The morphological organization of ependymoglia cells lining the ventricle in axolotls was examined using light and electron microscopic techniques. Circular‐shaped sacs were observed on the apical surface of ependymoglia cells in Neo and Met group axolotls. The product detected on the apical surface of the cells lining the ventricle was determined to contain mucopolysaccharide when stained with alcian blue on paraffin sections.

Mucopolysaccharides, or glycosaminoglycans (GAGs), present in the cell membrane and extracellular matrix of animals, play crucial roles in organogenesis, growth regulation, cell adhesion, signal transduction, inflammation, and interactions with pathogens (Rowlands et al. 2015; Zhang et al. 2009). Research into neurodegenerative diseases has included investigations into the biological effects and level disparities of GAGs, with suggestions that these might potentially serve as therapeutic agents.

Alcian blue is known to stain both sulfated and carboxylated acid mucopolysaccharides, as well as sulfated and carboxylated sialomucins (glycoproteins) (Demirbağ et al. 2015). In the present study, we observed ependymoglia arranged around the ventricle with mucopolysaccharide sacs on their apical surfaces and smaller secretory products on their basal surfaces. In order to delineate these cells still further, we conducted ultrastructural examinations with electron microscopy analysis.

Histological and ultrastructural analyses revealed that the light and electron microscopic features of these ependymoglia cells, bearing sacs at their apex, resemble goblet cells. At the alcian blue staining examination, these cells stained blue, similarly to goblet cells, and exhibited structural characteristics typical of goblet cells under electron microscopy. Situated in the ventricle wall of the axolotl brain, these goblet‐like cells may produce GAGs for cell proliferation, differentiation, and regeneration without scarring, potentially contributing to brain ventricle development and playing a role in cerebrospinal fluid flow. This organizational arrangement in the cellular structure suggests the possibility of a transport process occurring from basal to apical or vice versa within the cell.

Horstmann (1954) employed the term “tanycytes,” derived from “tanycyte,” meaning “stretched cells,” to describe ependymoglia cells that extend their cellular extensions over considerable distances (Reichenbach and Bringmann 2020). These contribute to the blood‐brain barrier with tight junctions in regions such as the median eminence and circumventricular organ region (Reichenbach and Bringmann 2020). These cells are known to be involved in the production of new neurons in adult hypothalamic neurogenesis (Lee et al. 2012). Tanycytes express neural stem cell markers such as nestin, components of the notch pathway, and hypothalamic progenitor‐specific transcription factors such as Rax (Berg et al. 2010; Zhao et al. 2003). Typically, tanycytes form a very thin layer in specific areas of adult mammalian brain tissue, including the wall of the diencephalic third ventricle, the dorsal and ventral walls of the mesencephalon, the floor of the fourth ventricle, and the ventral part of the spinal central canal. Additionally, tanycyte cells have been identified in the lateral ventricles of adult mice (Doetsch et al. 1997).

The goblet‐like cells observed in the telencephalon region of the axolotl may potentially perform functions similar to those of tanycyte cells in the central nervous system of vertebrates. These cells, exhibiting distinct granule contents as observed in the present study, may perhaps perform similar functions to those of tanycyte cells. Further comprehensive studies are now required to elucidate the functions and fine structures of these cells in light of their complex features.

The cell nucleus comprises a fibrillar structure known as chromatin, which exists in two distinct forms, euchromatin and heterochromatin (Carlberg and Molnár 2019). Cells actively synthesizing proteins typically contain abundant euchromatin, whereas those not engaged in protein synthesis primarily exhibit heterochromatic nuclei (Ross and Pawlina 2010). Brain development progresses through three main stages, neurogenesis, migration, and synaptogenesis (Aoki and Erisir 2014). Heterochromatin plays a supportive role in the cell migration process by affecting both the mechanical properties of the nucleus and the genetic processes occurring within it. Cell migration is a vital process in both health and disease, serving as a fundamental process in embryogenesis and the normal function of various tissues and systems in animals. Condensed chromatin and a stiffer nucleus can enhance nuclear resilience to migration stress and substantially reduce DNA damage during the migration process. Furthermore, the reorganization of heterochromatin in migrating cells is crucial for inducing migration‐specific transcriptional programs while inhibiting numerous other unnecessary transcriptional changes. Examinations of heterochromatic nuclei are, therefore, of particular importance in cell migration studies. Recent studies have demonstrated a marked increase in heterochromatin levels in the cell nucleus following stimulation of cell migration in various cell types (Maizels et al. 2017; Nair et al. 2019).

Our electron microscopic examination of Neo and Met group axolotl neurons revealed a greater abundance of euchromatic areas in the nuclei of Neo group neurons and of heterochromatic areas in the nuclei of Met group neurons. The nuclei of Met group telencephalon neurons contained large and numerous concentrated heterochromatic regions. Based on these findings, it maybe inferred that neuronal DNA becomes inactive post‐metamorphosis and that cell metabolism enters a passive state. Additionally, the transition from the neotenic stage to the metamorphic stage in axolotls appears to entail a change in chromatin organization within the nuclei, from euchromatic to heterochromatic, thus playing a pivotal role in the cellular migration process.

In previous studies, the presence of myelinated nerves in the anterior telencephalon region of the neotenic axolotl brain remained elusive using light microscopic methods (Lazcano et al. 2021). However, in the present study, we successfully demonstrated the presence of small‐diameter myelinated nerve fibers in the anterior telencephalon by using transmission electron microscopy. However, despite this observation, myelinated nerve fibers positively labeled with anti‐MBP could not be detected using immunohistochemical methods due to their small size. Metamorphosis was observed to involve changes in the morphology of axolotl myelinated nerve fibers. These morphological alterations may potentially be associated with the processes of aging with demyelination or of development with remyelination (Chen et al. 2020; Jennings and Carroll 2015).

By analyzing nerve cell extensions and histochemical determinations of catecholamines in brain structures, researchers have identified 17 distinct neuron groups (Sukhorukova et al. 2014). Catecholaminergic neurons in the brain are categorized into three types, dopaminergic, noradrenergic, and adrenergic (Carruth and Shahbazi 2015; Terry et al. 2023; Sukhorukova et al. 2014). The absence of neuromelanin in catecholaminergic neurons of certain laboratory animal species highlights its significance in the context of comparative neurobiology. While neuromelanin is present in the neurons of horses, sheep, dogs, giraffes, and frogs, it is absent in mice and rats, commonly used animals in laboratory settings. Notably, neuromelanin is observed in both the adult and tadpole stages in frogs (Sukhorukova et al. 2014; Zucca et al. 2004). Studies of the catecholaminergic system in salamanders have suggested that dopaminergic neuron populations in the diencephalon and mesencephalon are functionally analogous to the mammalian ventral tegmentum and substantia nigra (Parish et al. 2007). Furthermore, the presence of catecholaminergic neurons has been demonstrated in the axolotl spinal cord. These neurons are located in the ependymal layer lining the inner surface of the central canal, with apical extensions reaching the lumen of the canal and axonal extensions reaching from the cell body to the ventral median septum (Sims 1986). Additionally, dopaminergic neurons, a subset of catecholaminergic neurons, have been identified in the axolotl brain in previous studies (Berg et al. 2011; Parish et al. 2007).

In the present study, cells containing pigments, which we suspected to be neuromelanin, were detected in both paraffin and resin sections in the telencephalon and diencephalon regions of the axolotl brain. Even in unstained paraffin sections, these granules appeared dark brown in color. We hypothesize that these pigment‐containing cells in the axolotl brain may represent catecholaminergic neurons containing neuromelanin. Further research is now warranted to determine the specific subgroup to which these cells belong and to assess their distribution within neotenic‐metamorphic brain tissue.

## 5 Conclusion

This study investigated the effects of induced metamorphosis on the axolotl brain, focusing on the telencephalon region. Using immunohistochemical and stereological techniques, we analyzed the distribution of developing, maturing, and differentiating neurons, as well as the morphological changes occurring in brain tissue structure. Comprehensive histological, stereological, and ultrastructural examinations revealed significant alterations in cell structures and organization within the telencephalon when metamorphosis was induced in neotenic axolotls. These findings provide important insights into the cellular and ultrastructural morphology of the axolotl brain during metamorphic transition. The observed changes highlight the remarkable capacity of axolotls to adapt their neuronal and glial architecture. Moreover, this study contributes to the broader understanding of brain development, offering potential parallels to processes in other vertebrate systems, including mammals. However, to fully understand the mechanisms driving these changes, further investigation into the molecular pathways involved and their relationship to regenerative and developmental biology is essential.

## Author Contributions

A.A.K., G.Ö., S.B., and İ.K. designed or supervised the study and experiments. S.B. carried out the process of inducing the axolotls to metamorphosis. A.A.K. performed all experiments and performed imaging and analysis. İ.K. managed the project, providing funding for the study. A.A.K. prepared the draft of the article under the guidance of İ.K. and G.Ö. All authors read and edited the manuscript and approved the final version.

## Ethics Statement

This study protocol was reviewed and approved by the Istanbul Medipol University animal experiments local ethics committee, number E‐38828770‐772.02‐49005.

## Conflicts of Interest

The authors declare no conflicts of interest.

### Peer Review

The peer review history for this article is available at https://publons.com/publon/10.1002/cne.70031.

## Supporting information




**Supplement Figure 1**: MBP immunohistochemistry staining of rat brain. Myelinated axons to the encircled area are markedly anti‐MBP positive.

**Supplement Figure 2**: Neuromelanin. (a) Deparaffinized paraffin sections with no staining revealed dark‐colored granules in cells. (b,c) Paraffin sections after hematoxylin and eosin staining, arrows showed the dark colored granules.

**Supplement Figure 3**: Goblet‐like cell. Secretory sacs were encircled in a semi‐thin section stained with toluidine blue. The ultrastructure of the goblet‐like cell was shown. The contents of the sacs were not preserved.

**Supplement Figure 4**: Scanning electron microscope images of the diencephalon of the neotenic axolotl brain. (c,d) There is a thick covering layer on the surface of ependymoglia cells facing the ventricle (arrows). (e, f) When viewed from the apical surface of ependymoglia cells, cells have cilia (arrowhead).

**Supplement Figure 5**: Hypothalamus (a) The higher magnification of the dashed rectangle is shown in b,c. (b,c) Cilia on the surface of ependymoglia cells facing the ventricle and secretory sacs (arrowhead) surrounded by cilia are seen. (d) The cell surface (arrow) where the secretion occurs and the secreted product (arrowhead, blue labeled) are seen.

## Data Availability

The data that support the findings of this study are available from the corresponding author upon reasonable request.
