# Supplementary figures and images for: Investigating Neotenic and Metamorphic Axolotl Brain Complexity: A Stereological and Immunohistochemical Perspective
Source: J Comp Neurol. 2025 Mar 20;533(3):e70031. doi: 10.1002/cne.70031 (PMC11923732; doi:10.1002/cne.70031)

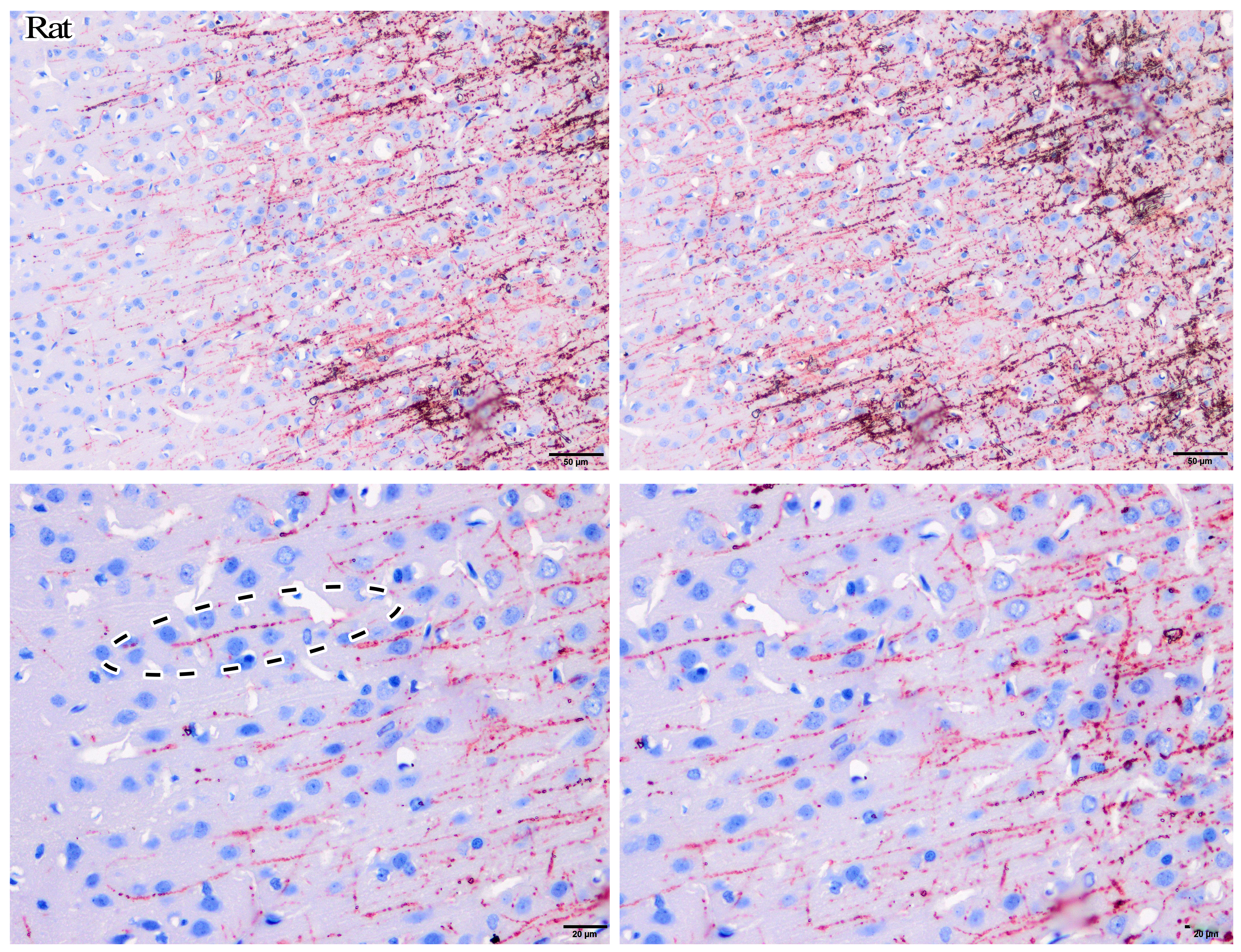

Supplement: Supplementary file 1 — Supplement Figure 1: MBP immunohistochemistry staining of rat brain. Myelinated axons to the encircled area are markedly anti‐MBP positive. [file CNE-533-e70031-s003.jpg]

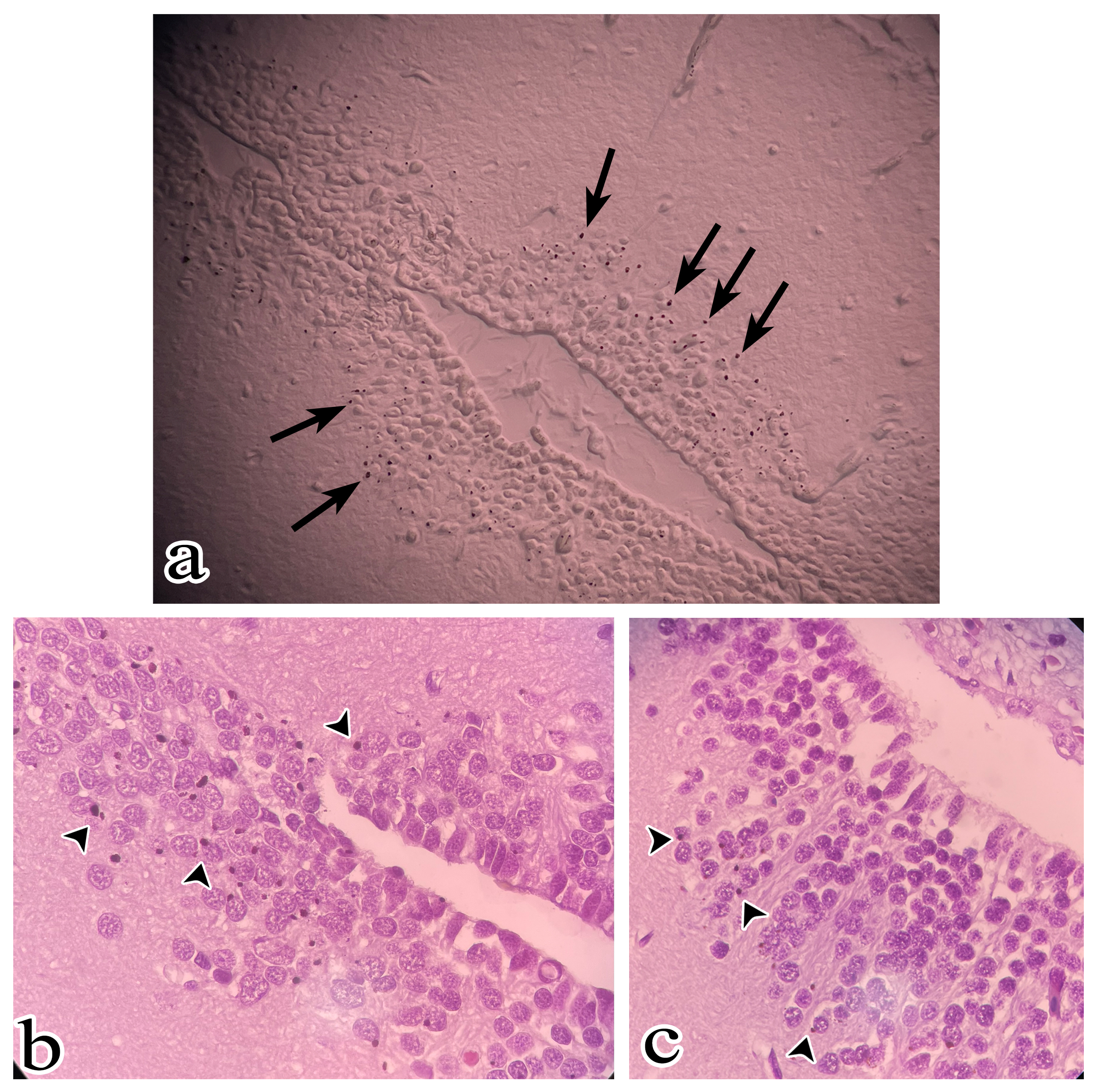

Supplement: Supplementary file 2 — Supplement Figure 2: Neuromelanin. (a) Deparaffinized paraffin sections with no staining revealed dark‐colored granules in cells. (b,c) Paraffin sections after hematoxylin and eosin staining, arrows showed the dark colored granules. [file CNE-533-e70031-s002.jpg]

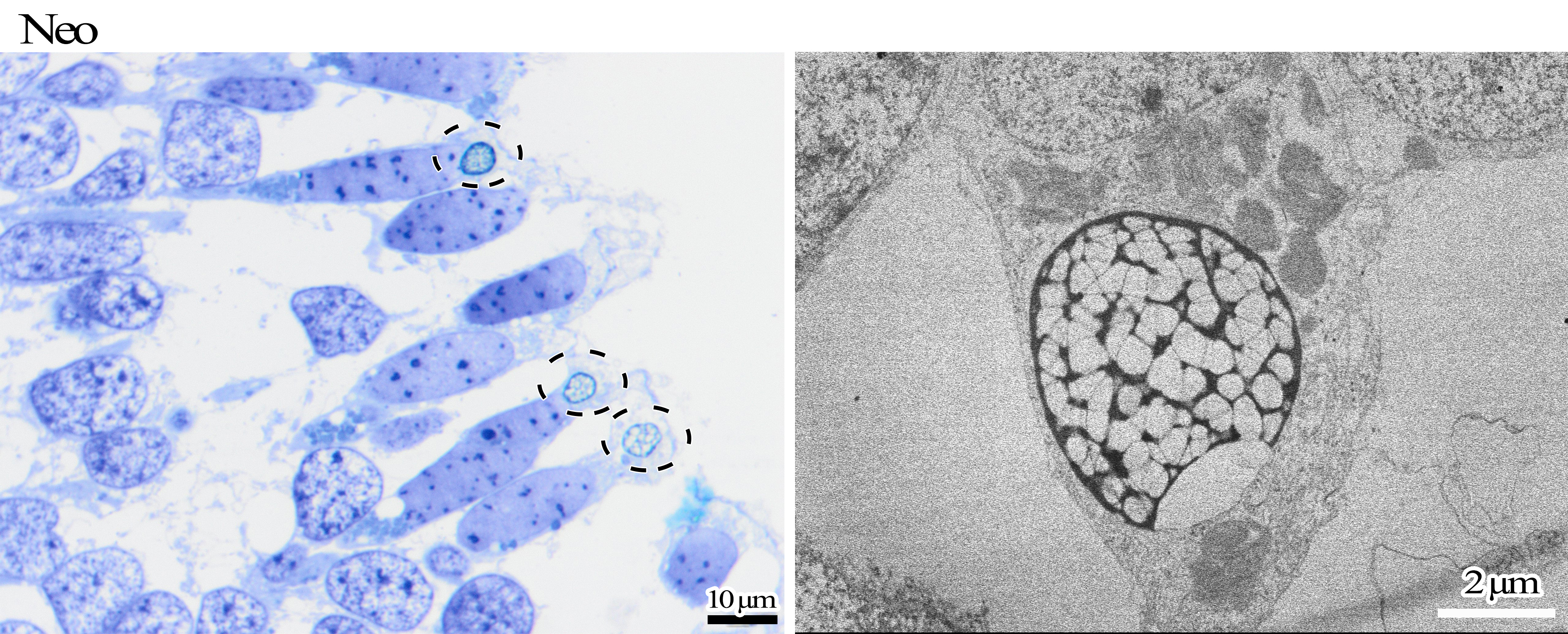

Supplement: Supplementary file 3 — Supplement Figure 3: Goblet‐like cell. Secretory sacs were encircled in a semi‐thin section stained with toluidine blue. The ultrastructure of the goblet‐like cell was shown. The contents of the sacs were not preserved. [file CNE-533-e70031-s005.jpg]

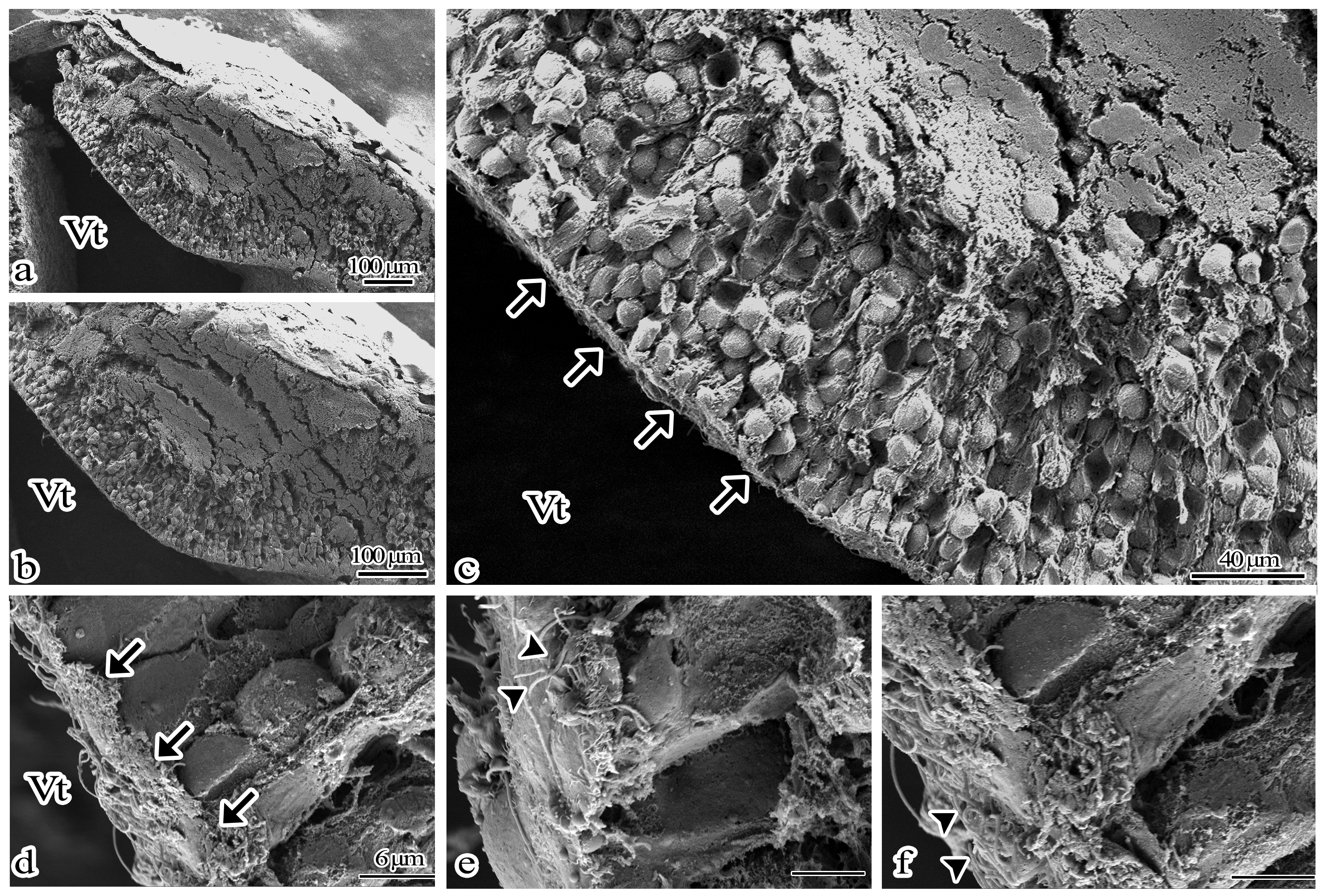

Supplement: Supplementary file 4 — Supplement Figure 4: Scanning electron microscope images of the diencephalon of the neotenic axolotl brain. (c,d) There is a thick covering layer on the surface of ependymoglia cells facing the ventricle (arrows). (e, f) When viewed from the apical surface of ependymoglia cells, cells have cilia (arrowhead). [file CNE-533-e70031-s004.jpg]

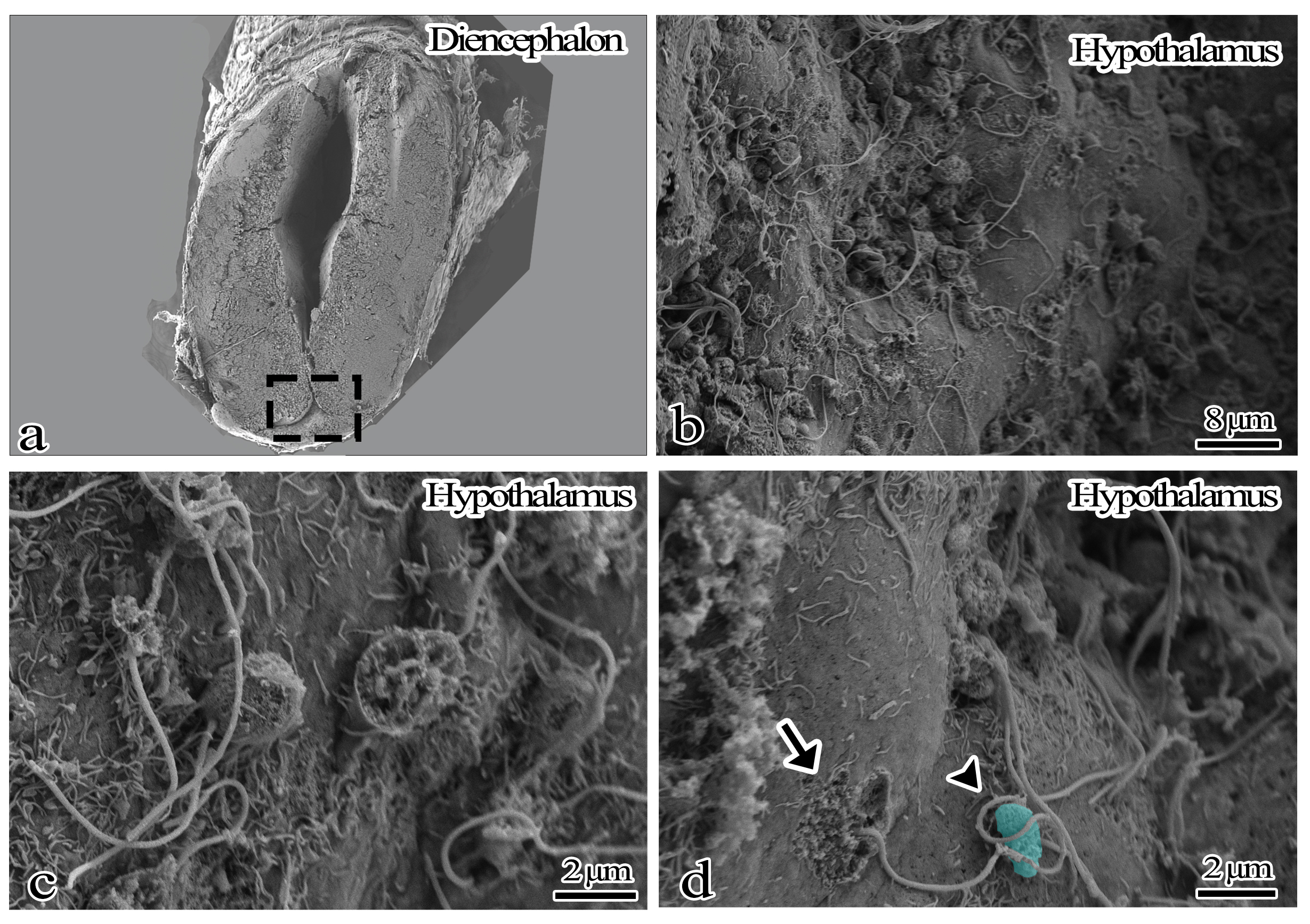

Supplement: Supplementary file 5 — Supplement Figure 5: Hypothalamus (a) The higher magnification of the dashed rectangle is shown in b,c. (b,c) Cilia on the surface of ependymoglia cells facing the ventricle and secretory sacs (arrowhead) surrounded by cilia are seen. (d) The cell surface (arrow) where the secretion occurs and the secreted product (arrowhead, blue labeled) are seen. [file CNE-533-e70031-s001.jpg]
